# A Review of Vision-Based Pothole Detection Methods Using Computer Vision and Machine Learning

**DOI:** 10.3390/s24175652

**Published:** 2024-08-30

**Authors:** Yashar Safyari, Masoud Mahdianpari, Hodjat Shiri

**Affiliations:** 1 Civil Engineering Department, Faculty of Engineering and Applied Sciences, Memorial University of Newfoundland, St. John’s, NL A1B 3X7, Canada; ysafyari@mun.ca (Y.S.); hshiri@mun.ca (H.S.); 2 Department of Electrical and Computer Engineering, Memorial University of Newfoundland, St. John’s, NL A1C 5S7, Canada; 3 C-CORE, 1 Morrissey Rd, St. John’s, NL A1B 3X5, Canada

**Keywords:** pothole detection, computer vision, image processing, machine learning, deep learning, target detection, convolutional neural networks

## Abstract

Potholes and other road surface damages pose significant risks to vehicles and traffic safety. The current methods of in situ visual inspection for potholes or cracks are inefficient, costly, and hazardous. Therefore, there is a pressing need to develop automated systems for assessing road surface conditions, aiming to efficiently and accurately reconstruct, recognize, and locate potholes. In recent years, various methods utilizing (a) computer vision, (b) three-dimensional (3D) point clouds, or (c) smartphone data have been employed to map road surface quality conditions. Machine learning and deep learning techniques have increasingly enhanced the performance of these methods. This review aims to provide a comprehensive overview of cutting-edge computer vision and machine learning algorithms for pothole detection. It covers topics such as sensing systems for acquiring two-dimensional (2D) and 3D road data, classical algorithms based on 2D image processing, segmentation-based algorithms using 3D point cloud modeling, machine learning, deep learning algorithms, and hybrid approaches. The review highlights that hybrid methods combining traditional image processing and advanced machine learning techniques offer the highest accuracy in pothole detection. Machine learning approaches, particularly deep learning, demonstrate superior adaptability and detection rates, while traditional 2D and 3D methods provide valuable baseline techniques. By reviewing and evaluating existing vision-based methods, this paper clarifies the current landscape of pothole detection technologies and identifies opportunities for future research and development. Additionally, insights provided by this review can inform the design and implementation of more robust and effective systems for automated road surface condition assessment, thereby contributing to enhanced roadway safety and infrastructure management.

## 1. Introduction

In the current world, the quality of road infrastructure plays a crucial role in facilitating economic activities, social interaction, and public safety [1]. However, factors like heavy traffic, severe weather conditions, and natural disasters can compromise the durability and integrity of roadways [2]. Structural damage, such as cracks and potholes, endangers road users and increases maintenance costs [3,4].

Potholes are typically defined as rough, uneven depressions on the road surface resulting from structural failures due to water infiltration and vehicular pressure [5]. These road defects are widespread and pose significant safety risks to motorists [6]. Factors such as climate change and the use of substandard materials by some construction companies have been identified as key contributors to the formation of potholes [7].

Potholes can significantly impact the economy by increasing vehicle maintenance costs, fuel consumption, travel time, and reducing tire longevity [8]. Additionally, they negatively affect the environment by raising the emissions of greenhouse gases, such as carbon dioxide [7]. Consequently, addressing structural damage risks and implementing appropriate measures to improve and maintain road infrastructure is essential [9]. This action will ensure the safety and functionality of roadways, promoting sustainable mobility and reducing economic and environmental costs [10].

Early detection of potholes is critical for enhancing driver safety and reducing accidents. Manual visual inspection is the most commonly used method for detecting potholes, performed by structural engineers and certified inspectors [11]. Recently, many countries have allocated resources and budgets for pothole detection and repair [12]. For example, the United Kingdom (UK) government has announced plans to spend over GBP 5 billion on pothole identification and repair from now until 2025 [7]. Similarly, in San Diego, United States (US), more than 30,000 potholes were repaired annually, with residents encouraged to report potholes to alleviate the burden on local road maintenance agencies. However, relying on non-automatic detection techniques is subjective, as it depends on engineers’ and inspectors’ personal judgment and experience [13]. These traditional methods can be time-consuming, costly, and inefficient [14].

Road agencies can implement advanced computer vision detection systems to address these challenges to reduce maintenance costs, increase road safety, and ensure adequate road upkeep [14]. The importance of rapid detection and repair has prompted the development of various pothole detection techniques [15]. In recent years, computer vision and machine learning have been used for automated surface fault detection, providing a more precise and efficient method for maintaining road infrastructure [16]. Moreover, pothole identification has become integral to advanced driver-assistance systems (ADASs) in L3/L4 autonomous vehicles [17].

Automakers are integrating pothole detection technologies into autonomous driving systems, underscoring the need for improved road maintenance [17]. For instance, Jaguar Land Rover has developed data-driven technology to inform drivers of pothole locations. ClearMotion has created an intelligent suspension system designed to anticipate, absorb, and counteract shocks and vibrations caused by road potholes [18]. These innovations highlight the growing importance of efficient pothole detection and repair in ensuring road safety and autonomous driving technology advancement [19].

This paper significantly contributes to advancing road infrastructure maintenance and safety by addressing the critical issue of potholes. It offers a comprehensive overview of cutting-edge pothole detection algorithms, categorizing them into four groups: traditional two-dimensional (2D) image processing, three-dimensional (3D) point cloud processing, machine learning/deep learning methods, and hybrid approaches.

**Figure 1:**
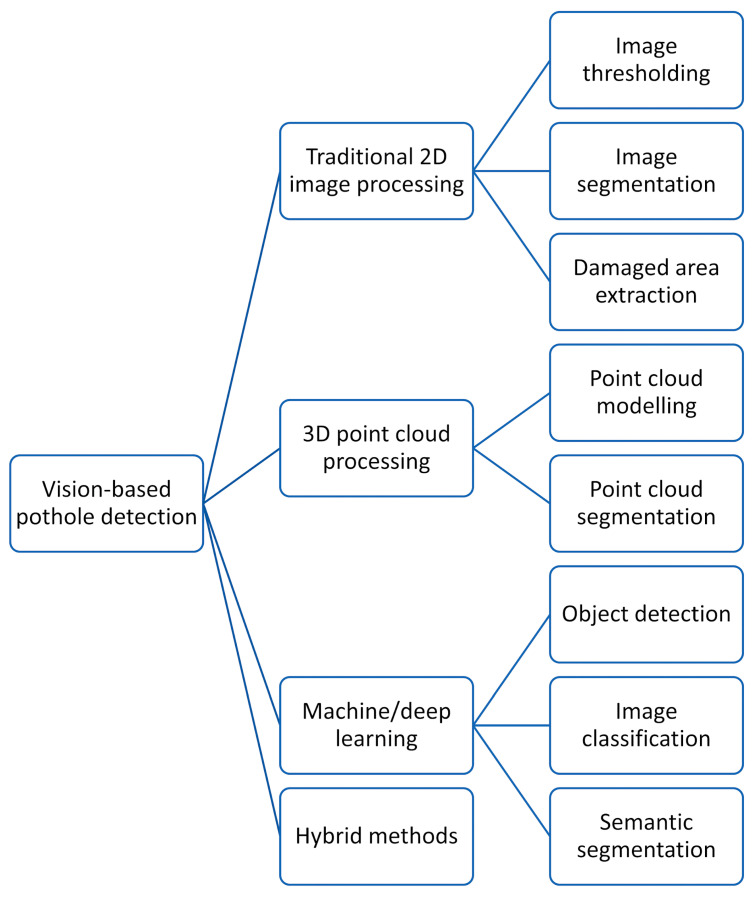
An overview of vision-based pothole detection algorithms.

In recent years, computer vision techniques have played a crucial role in obtaining 3D road data and detecting potholes [4], 3D point cloud processing, machine/deep learning methods, and hybrid approaches (see Figure 1). However, recent studies provide limited discussion on methods such as 3D point cloud processing and machine/deep learning data processing pipelines [20]. This study primarily focuses on computer vision-based pothole detection, categorized into four main areas: traditional 2D image processing [21], 3D point cloud processing [22], machine/deep learning [23], and hybrid approaches [11] (see Figure 1).

Traditional 2D image processing techniques for pothole detection include thresholding, segmentation, and identifying damaged regions. Methods like Otsu’s thresholding [24], histogram-based thresholding [25], morphological operations [26], and spectral clustering [27] are used to isolate damaged areas and extract potential pothole contours, enabling accurate detection of road defects [20]. Innovative 3D point cloud-based techniques utilize modeling and segmentation approaches for detecting road potholes [28]. These methods incorporate surface normal information into geometric modeling refinement, labeling, and clustering. Clustering and region-growing algorithms further enhance detection accuracy. Machine and deep learning techniques, such as deep convolutional neural networks (CNNs) [29], have revolutionized road pothole detection. These methods use large annotated road datasets to achieve accurate and automated pothole detection through image classification [30], object detection [31], and segmentation [32] tasks. Finally, hybrid methods combine classical 2D image processing with 3D point cloud modeling, segmentation, or machine/deep learning approaches [33]. These innovative approaches use multi-modal road data, such as images and 3D point clouds, to efficiently detect potholes. By integrating diverse data sources and algorithms, hybrid methods improve the accuracy and robustness of road defect detection.

In 2023, Saisree and Kumaran [15] explored the application of deep learning for pothole detection on both muddy and highway roads. Their study employed a classification approach using pre-trained models like ResNet50, InceptionV2, and VGG19. By comparing model performance metrics, such as accuracy, precision, and recall, they determined VGG19 to be the most effective in their experimental setup. Xu et al., (2023) proposed a Vision-IMU-based detection and ranging (VIDAR)-based approach for pothole detection, combining vision and IMU sensors. Their method focused on filtering, marking, and framing potholes on flat pavements using MSER for dimension estimation [4]. By comparing their method to a classical approach and evaluating performance metrics, they demonstrated an improvement in pothole detection accuracy compared to traditional monocular vision systems. More recently, Ruseruka et al. (2024) proposed a deep learning-based approach for real-time pothole detection and dimension estimation utilizing in-vehicle technologies. Their model, employing the YOLO algorithm, demonstrated high accuracy in identifying and measuring potholes [34]. By addressing the limitations of traditional manual surveys and expensive sensor-based methods, this research offers a cost-effective and efficient solution for road maintenance. In another study, Mahalingesh et al. (2024) presented a practical solution to the pothole detection problem by integrating the YOLOv8 object detection model with hardware components. Their work focused on real-time detection, utilizing a Raspberry Pi and Arduino Mega for processing and control. By achieving a respectable average precision and recall, the study demonstrated the feasibility of deploying such a system for improved road safety and reduced maintenance costs in a real-time system [35]. Sasank and Tallam (2024) also proposed a CNN-based pothole detection system utilizing the YOLO algorithm. Their research focused on real-time detection and dimension estimation of potholes. By training their model on a large dataset from scratch and achieving a high mean average precision of 92%, they demonstrated the potential of their approach for improving road maintenance efficiency and safety through early detection and assessment of pothole dimensions [36].

The purpose of this study is to review existing research, open-access datasets, and the challenges faced in the field. Highlighting its broad applicability and relevance to current research trends, this article highlights the importance of data sensing and processing for developing advanced pothole detection algorithms. Specifically, this review paper aims to (1) comprehensively categorize and compare the performance of diverse vision-based pothole detection techniques, including traditional 2D image processing, advanced 3D point cloud analysis, and cutting-edge machine and deep learning algorithms; (2) critically evaluate the effectiveness and limitations of various sensing systems employed for pothole detection in terms of accuracy, reliability, and cost-efficiency; (3) conduct a rigorous analysis of existing methodologies to identify their strengths, weaknesses, and potential biases; and (4) delineate promising avenues for future research and development in automated pothole detection systems, with a focus on enhancing detection accuracy, real-time performance, and adaptability to diverse road conditions. As such, this review addresses several gaps in the existing literature: the lack of comparative analyses between different pothole detection methods, insufficient evaluation of the performance of various sensing technologies, and the need for a comprehensive synthesis of traditional and modern approaches.

This article is structured as follows: Section 2 examines the stages and methodologies of pothole detection processing. Section 3 discusses essential aspects of pothole data collection, focusing on sensing technologies and data acquisition systems. Section 4 reviews diverse pothole detection approaches, including traditional image processing, 3D analysis, machine learning, and hybrid methods. Lastly, Section 5 offers concluding remarks and suggests future research directions in pothole detection.

## 2. Pothole Detection Processing Pipeline

**Figure 2:**
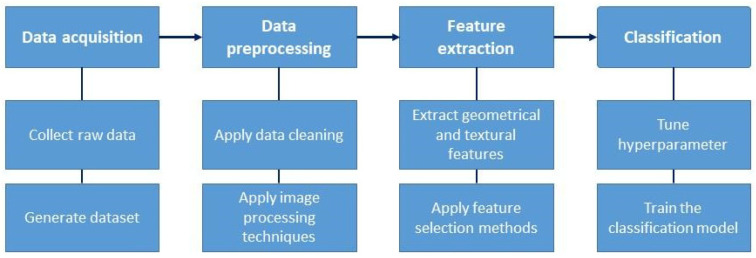
Pothole detection processing pipeline.

The pothole detection process involves several steps: data acquisition, preprocessing, feature extraction, and classification (see Figure 2). The data acquisition phase is at the core of this framework and operates as the cornerstone of pothole detection systems. This phase comprises two main steps: raw data collection and dataset generation, both crucial for capturing the diverse conditions of road surfaces, including potholes, to ensure accurate and reliable detection [37].

Historically, conventional methods primarily relied on 2D imaging techniques, such as cameras, to capture surface images [38]. However, these methods often fell short of accurately representing the complex spatial structures of road surfaces and were susceptible to environmental factors like poor lighting conditions [39]. To address these limitations, researchers have adopted advanced 3D imaging technologies [40].

Laser scanning, for example, uses trigonometric principles to provide exact 3D geometry information on road surfaces [41]. Despite its unmatched accuracy, laser scanning requires specialized equipment mounted on dedicated vehicles, making it less practical due to high costs and maintenance requirements [42]. Alternatively, initially designed for gaming applications, sensors like the Microsoft Kinect offer RGB cameras, infrared sensors, and motion-tracking capabilities, making them suitable for road imaging tasks [43]. However, challenges such as infrared saturation in direct sunlight can limit their effectiveness [44]. Other 3D imaging techniques, such as multi-view geometry and stereo vision, utilize multiple cameras to capture images from different perspectives, enabling accurate reconstruction of road surfaces [45]. Each technology presents unique advantages and limitations, but the overarching goal remains to capture precise and comprehensive road surface data essential for effective pothole detection [46].

The data preprocessing phase is a critical preparatory step in the pothole detection process, aimed at refining and enhancing the acquired raw data to prepare it for subsequent analysis [47]. This phase involves applying signal-processing techniques, such as data cleaning and image processing, to transform the dataset and improve its quality for analysis [48]. Data cleaning techniques are employed to eliminate noise, artifacts, and unwanted elements from the raw data, ensuring that the dataset is free from inconsistencies or errors that could affect the accuracy of the detection process [49]. These techniques may include filtering methods to remove high-frequency noise or outliers and interpolation techniques to fill in missing data points [50]. Additionally, image processing techniques are applied to enhance the clarity and quality of the acquired images, making them more suitable for feature extraction and classification [51]. These techniques may involve adjusting brightness and contrast, sharpening edges, or removing distortions to improve the overall visual appearance of the images [52]. By removing noise and enhancing image clarity, the dataset is optimized for more effective analysis during subsequent stages of the detection process [53].

In the context of pothole detection, data cleaning might involve the use of median or Gaussian filters to smooth out noise while preserving important edges that indicate surface defects [54]. Outlier detection methods, such as z-score analysis [54] or IQR filtering [55], can be applied to remove data points that deviate significantly from the norm, which are often caused by sensor errors or extraneous environmental factors. Image processing for clarity enhancement could include histogram equalization to improve contrast, morphological operations to remove small artifacts, and edge detection algorithms like Canny or Sobel to highlight the boundaries of potholes more clearly [56]. Techniques such as bilinear or bicubic interpolation can be used to estimate and fill missing pixel values, ensuring continuity in the data [57].

Next, in the feature extraction phase, the relevant characteristics indicative of potholes are precisely identified and isolated from the preprocessed data [26]. This phase is pivotal in transforming raw data into meaningful representations that facilitate accurate pothole identification [56]. During the feature extraction phase, the preprocessed data are comprehensively analyzed to determine patterns or attributes that distinguish potholes from other road elements [33]. Various features are considered, including texture patterns, color variations, geometric properties, and structural attributes characteristic of potholes [58]. Texture-based features involve analyzing surface irregularities or patterns typically associated with potholes, such as cracks or indentations. In contrast, color-based features focus on detecting contrasts or anomalies within the road surface that signify potholes [59]. Geometric features include shape descriptors or spatial arrangements that exhibit distinctive characteristics of potholes, such as circular or irregular shapes [60]. Additionally, structural features involve analyzing spatial relationships or connectivity patterns between road elements to identify potential areas of damage or deterioration [61]. The feature extraction process is imperative for training machine learning models or developing classification algorithms that can accurately detect potholes amidst varying road conditions [62]. Moreover, the selection of appropriate features significantly influences the performance of the detection system, as it determines the discriminative power and robustness of the employed algorithms [63]. Through a sufficient feature extraction phase, the pothole detection system can accurately distinguish potholes, thereby improving the overall accuracy and reliability of the detection process [64].

In addition to these features, advanced techniques like wavelet transform [56] can be employed to analyze the multi-scale texture of the road surface, capturing both fine and coarse details indicative of potholes [65]. For example, Local Binary Patterns (LBPs) [66] can be used for texture classification by encoding the local contrast information. For color-based features, techniques such as color histograms [12] or color moment invariants [4] help quantify color distributions and identify anomalies. Geometric feature extraction [67] might include methods like the Hough Transform [2,68] to detect specific shapes or contours that signify potholes [69]. Structural features could benefit from graph-based approaches, where nodes represent significant surface points, and edges denote spatial relationships, helping to detect patterns of deterioration [70]. By leveraging a combination of these techniques, the feature extraction phase can yield a robust set of attributes that enhance the accuracy and reliability of pothole detection algorithms.

The final step in the pothole detection process is classification, where specific algorithms are applied to the extracted features to determine the presence or absence of potholes within the dataset [26]. Classification algorithms use these features to differentiate between road defects, such as potholes and non-defective road surfaces [71]. Standard machine learning and deep learning techniques, such as support vector machines (SVMs), decision trees (DTs), and CNNs, are employed for classification tasks [72]. These algorithms learn from labeled training data to make predictions about the presence of potholes based on the extracted features [73]. However, achieving optimal performance requires fine-tuning hyperparameters and training the classification model on the dataset [74]. This process involves adjusting parameters such as learning rates, regularization strengths, and model architectures to enhance the model’s accuracy [75]. Selecting an optimal classification method and fine-tuning hyperparameters are critical for achieving accurate and reliable pothole detection [76]. Evaluating and comparing different classification algorithms and tuning hyperparameters helps identify the most suitable approach for the specific requirements and challenges of the detection task [77]. The classification model can effectively detect potholes through rigorous optimization and training, thereby contributing to improved roadway safety and infrastructure management [78]. In practice, this involves splitting the dataset into training, validation, and test sets to prevent overfitting and ensure the model generalizes well to unseen data [79]. Cross-validation techniques, such as k-fold cross-validation [80], are used to evaluate the model’s performance across different subsets of data. Fine-tuning hyperparameters might include grid search [81] or randomized search methods [82] to systematically explore different parameter combinations. Ensemble methods, like Random Forests [83] or Gradient Boosting [84], can be employed to improve classification accuracy by combining the predictions of multiple base learners [85]. For deep learning models like CNNs [86], steps such as dropout, batch normalization, and data augmentation are critical to enhancing model robustness and preventing overfitting [87]. Additionally, interpretability methods such as SHAP values [88] or Grad-CAM [89] can be used to understand the model’s decision-making process, ensuring transparency and trustworthiness in pothole detection outcomes. By iteratively refining these models, the system can achieve high accuracy and reliability, making significant contributions to roadway safety and maintenance efficiency.

## 3. Pothole Data Collection

### 3.1. Sensors and Systems

Collecting a substantial and diverse dataset is essential to study and analyze potholes effectively. This stage involves using cameras, laser scanners, or specialized devices to capture images or sensor readings representing various road surfaces and conditions, including potholes [21].

**Figure 3:**
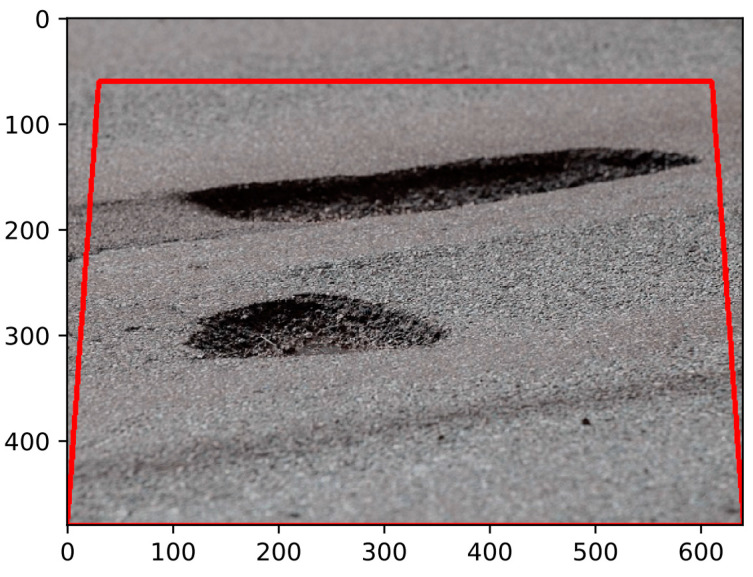
An example of a 2D road image [92].

For accurate road surface assessment, pothole detection relies on sensors and imaging technologies [90]. Initially, 2D imaging techniques were used, but their limitations in explicitly illustrating spatial structure, such as the inability to measure depth accurately and their vulnerability to factors such as poor illumination, which can lead to inaccurate readings, made them less effective [91]. Figure 3 demonstrates an example of a 2D road image.

**Figure 4:**
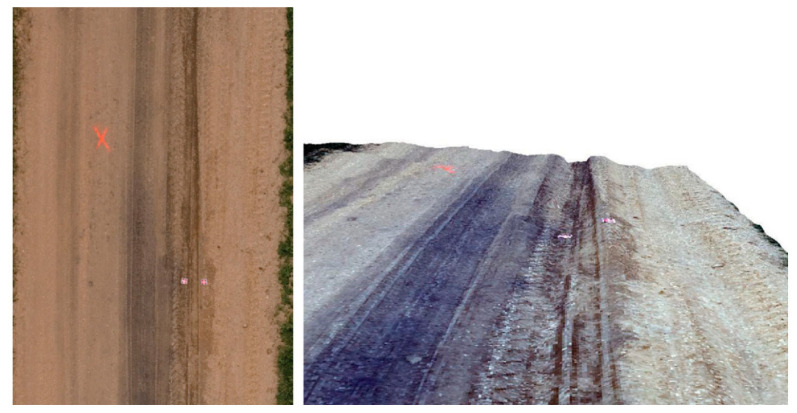
An example of a 3D road image [105].

To improve accuracy and enhance road damage detection, researchers have turned to 3D imaging technologies [93]. In addition, alternative 3D imaging techniques include multi-view geometry and using either a single movable camera [94] or a series of synchronized cameras [95] to capture images. These methods rely on dense correspondence matching between road images, employing techniques such as structure from motion (SfM) [96] for monocular dynamic correspondence matching and stereo vision for binocular depth estimation [32,97]. While digital cameras are cost-effective, poor illumination can compromise their accuracy [98]. Despite these challenges, other 3D reconstruction methods, such as shape from focus (SFF) [99,100,101], shape from shading (SFS) [102], and time-of-flight (ToF) [103,104], offer alternatives for acquiring 3D geometry but are less commonly used for road data gathering [93]. These technologies aim to capture precise and comprehensive road surface data to enhance pothole detection and damage assessment. Figure 4 shows an example of a 3D road image collected by an unmanned aerial vehicle (UAV) system.

Based on trigonometric triangulation principles, laser scanning is a well-established technology for acquiring precise 3D road geometry information [93]. Despite their precision, laser scanners require mounting on dedicated road inspection vehicles, making them less practical due to high equipment costs and maintenance complications [22].

Initially developed for gaming purposes, Microsoft Kinect sensors are also helpful in road imaging due to their RGB cameras, infrared sensors, and motion-tracking capabilities [93]. However, challenges such as infrared saturation in direct sunlight, which can lead to inaccurate readings, can limit their efficacy for collecting continuous road data [106].

### 3.2. Public Datasets

Several open-access road pothole detection datasets were introduced in previous studies for the development of the supervised algorithm. For instance, Abhinav Kulshreshth’s pothole detection dataset combines images sourced from Google and Kaggle, divided into train (1167 images), validation (108 images), and test (136 images) folders, containing both normal and pothole images. This dataset is publicly available on the Kaggle website. Another dataset by Viren Baraiya includes 367 color images of healthy roads and 357 images of roads with potholes in the training set, with eight images per category in the test set. Similar to the previous dataset, this dataset is available through the Kaggle website. The Pothole-600 dataset [17] provides color images and transformed disparity images generated using stereo-matching algorithms [107], facilitating research on road surface defects. This dataset is publicly available at Google.

Additionally, a dataset focused on semantic segmentation of potholes and cracks offers 4340 image–mask pairs for training, validation, and testing purposes [108]. This dataset was divided into training, validation, and test datasets, with 3340, 496, and 504 images making up 77, 11, and 12 percent of the images in each set, respectively. In this study, the dataset was trained in neural networks for semantic segmentation in the SHREC2022 competition, resulting in videos and images. This dataset can be accessed through the DeepLearning website. Another large-scale dataset was created to detect potholes at the instance level. A training set, a test set, and an annotation CSV file comprise this dataset. There are 2658 color images of healthy roads and 1119 color images of potholed roads in the training set. In addition, 628 color images were used in the test set. A GoPro Hero 3+ camera was employed to capture these images, which can be found on the Kaggle website.

In another study, Liu et al. (2024), generated a comprehensive dataset to support multiple-type distress detection in asphalt concrete pavement. It includes five types of images: visible, infrared, and three fusion images with varying blends of visible and infrared content. The dataset also addresses five distress types: longitudinal cracking, transverse cracking, fatigue cracking, edge cracking, and potholes. A total of 3213 images with a resolution of 640 × 480 were used, stratified into training (64%), validation (16%), and test (20%) subsets. This dataset is pivotal for the training and evaluation of CNN object detection models and aims to enhance the accuracy and reliability of pavement distress detection using both traditional and advanced imaging techniques [16]. The next pothole detection dataset was generated for object detection with 3033 color images as a training dataset, 491 color images as a validation dataset, and 246 color images as a test dataset. This dataset has been considered one of the largest datasets in this field, and several object detection YOLO (You Only Look Once) models such as YOLO version 5 (YOLOv5), YOLOv7, and YOLOv8 have been trained and validated on this dataset. This dataset is available on the Universe website. Furthermore, semantic segmentation datasets of Indian roads include 2475 training images and 752 test images annotated for road, pothole, footpath, shallow path, and background classes. Lastly, a dataset from Japan offers 9053 road images capturing 15,435 instances of road damage using a smartphone-mounted car setup, which is crucial for road damage detection research (available at the GitHub website [109]. Table 1 summarizes the key details of the public datasets introduced in this review paper.

## 4. Pothole Detection Approaches

Computer vision-based algorithms have been developed to detect potholes on roads, and these algorithms can be categorized into distinct categories. Traditional 2D image processing approaches use explicit programming techniques to enhance, compress, transform, threshold, and segment road RGB or disparity/depth images [110]. In contrast, machine/deep learning-based algorithms employ state-of-the-art CNNs to detect road potholes using object detection, image classification, or semantic segmentation techniques [111]. Another approach involves 3D road point cloud processing-based methods, which fit geometric models such as planar or quadratic surfaces to observed road point clouds and then segment them by comparing the observations with the fitted surfaces [112]. Additionally, hybrid approaches offer a promising avenue for enhancing system performance by combining the strengths of multiple algorithmic techniques. By integrating image processing, computer vision, and machine learning, these systems can effectively address the challenges posed by varying road conditions, lighting, and occlusion. These methods often involve pre-processing images, extracting relevant features, and employing deep learning models for classification and detection. Additionally, incorporating 3D point cloud data can provide valuable depth information, enhancing the accuracy and robustness of the system. Sensor fusion is another key component of hybrid approaches, as it allows for the combination of data from multiple sensors, such as cameras, LiDAR, and radar. This integration provides a more comprehensive understanding of the road environment, leading to improved detection accuracy and reliability. Hybrid methods offer several advantages, including increased accuracy, robustness, and adaptability to different road conditions. However, challenges such as data fusion, computational complexity, and sensor calibration must be carefully addressed to ensure optimal performance [113].

### 4.1. Traditional 2D Image Processing

Traditional 2D image processing in pothole detection studies, while effective in many cases, does have its limitations. It can generally be divided into three groups: image thresholding, image segmentation, and damaged area extraction. This methodology involves detecting and segmenting damaged road areas using histogram-based algorithms [21] such as Otsu’s thresholding [114], triangle thresholding [21], and adaptive thresholding [115,116] for grayscale road image segmentation [117]. Subsequent processing involves median filtering [118] and morphology operations [119] to reduce noise. Pixel intensity histograms are then used to identify damaged road regions. However, poor illumination conditions often compromise the accuracy of traditional segmentation techniques based on color or grayscale images, highlighting the need for more advanced approaches.

Several approaches have been developed to mitigate this problem, using Microsoft Kinect sensors and highly accurate laser scanners to segment depth or disparity images [11,106,120]. Using these sensors, researchers have used depth images to perform more effective segmentation using algorithms such as wavelet transforms [121] or watershed methods [122] to differentiate between damaged and undamaged road surfaces. For instance, watershed-based real-time algorithms were presented by Chung and Khan (2019) [92] to detect multiple potholes on asphalt roads. A threshold value in the inverted color space of the image was determined using the inverted binary and Otsu thresholding techniques. A morphological technique employing open and closed kernels is applied to reduce small noises and emphasize prominent pothole edges. Before applying the watershed algorithm, the algorithm used distance transform to identify markers on the pre-watershed image. The proposed algorithm performed at approximately 33.1 frames per second (fps) in real-time based on extensive testing. The experimental results demonstrated that the algorithm could detect potholes of various sizes and structures across various roads, including smooth, aged, and degraded ones. The presented watershed-based approach offers a versatile method for detecting multiple potholes on asphalt roads using image sensors, showcasing its adaptability and effectiveness.

In another study, Buza et al. (2013) [27] introduced a novel unsupervised vision-based method for detecting potholes, which addressed the problem of planning asphalt pavement rehabilitation and repairs. In contrast to prior methods that usually require expensive equipment, additional filtering, and training phases, the proposed approach used image processing and spectral clustering, eliminating the need for expensive equipment, additional filtering, and training phases. After detecting frames with defects, the method analyzed histogram-based data from grayscale images using spectral clustering to identify regions. This method can use commonplace equipment to identify potholes, estimate their surfaces, and provide rough estimates while remaining cost-effective. As a result of testing various pothole images, the proposed method reported an accuracy of 0.81 in surface estimation, demonstrating the effectiveness of their approach in detecting potholes with reasonable accuracy and presenting a good solution to pavement maintenance planning that is practical and cost-effective.

Using 2D image analysis, Ryu et al. (2015) proposed a novel method for detecting potholes that improved the efficiency and accuracy of previous methods [117]. The proposed method included three key steps: segmentation, candidate region extraction, and decision-making. In the initial steps, histogram analysis and morphology filtering methods were used to segment and extract dark regions within asphalt images that may indicate potholes. Candidate regions were isolated based on size and compactness to refine the detection process. A decision was made by comparing pothole-specific characteristics with background attributes, allowing for accurate detection of potholes amidst similar patterns or irregularities in the pavement. Based on a database of 2D asphalt images collected from national highways in South Korea, with and without potholes, this method demonstrated superior performance over existing methods. This technique achieved an overall accuracy of 0.91, precision of 0.85, and recall of 0.93, significantly outperforming the previous method’s accuracy of 0.71, precision of 0.70, and recall of 0.61. This method used multiple image features to make pothole detection systems more reliable and efficient.

As part of researchers’ efforts to address the critical need for accurate road network maintenance, Wang et al. (2017) [123] presented an innovative method for detecting and segmenting potholes on asphalt pavement surfaces. Using a wavelet energy field, this approach efficiently integrated grayscale and texture information to detect pavement potholes. The method involved two fundamental processes: first, morphological processing and geometric criteria were used to construct the wavelet energy field of pavement images for pothole detection; second, a Markov random field model was employed to segment potholes and extract their edges. The method was tested using 120 pavement images, and the Matrix Laboratory (MATLAB-R2016b) prototype demonstrated its ability to distinguish potholes from other road anomalies, such as cracks, patches, greasy dirt, shadows, and maintenance hole covers. The proposed method achieved an overall detection accuracy of 0.86, with 0.83 precision and 0.87 recall, outperforming other existing methods. Despite some limitations related to specific pavement conditions, this method provided valuable knowledge about road maintenance strategies and enhanced the reliability of pothole detection systems.

Roads are highly distinguishable from the damaged areas of the road when the disparity transformation transforms them. This type of image produces a closed-form solution to the energy minimization problem, reducing the need for iterative optimization computations. The geometric structure of road surfaces can be depicted by depth/disparity images, which can be more informative when detecting potholes. For instance, a novel road damage detection algorithm based on unsupervised segmentation of disparity maps was developed by Fan and Liu (2020) [11]. This algorithm transforms a disparity map using a stereo rig roll angle and a road disparity projection model to minimize an energy function. Instead of relying on nonlinear optimization techniques such as golden section search and dynamic programming, the authors directly derived a numerical solution for the energy minimization problem [124,125]. It is possible to extract damaged road areas by segmenting the disparity map following the transformation with Otsu’s thresholding method. This algorithm is notable for not requiring any parameters during road damage detection. It has been demonstrated that the algorithm was highly accurate at the pixel level, with an accuracy of approximately 0.97. The authors provided the algorithm’s source code, highlighting its accessibility. In addition to improving road damage detection accuracy and efficiency, this innovative approach was used in real-time scenarios and even vehicle state estimation, demonstrating its versatility and significance as a transportation technology solution. As a result of the method’s ability to automate the labeling of training data, its value in advancing road damage detection methodologies was enhanced.

Based on road disparity map estimation and segmentation, Fan et al. (2022) [32] presented an innovative and efficient algorithm for detecting road potholes, addressing the common problem of potholes causing road damage. As a result of including the stereo rig roll angle in the shifting distance calculation, perspective transformation accuracy was enhanced while computational complexity was minimized. In order to better detect damaged road areas, semi-global matching was used to estimate dense subpixel disparity maps, followed by disparity map transformation. Using the Simple Linear Iterative Clustering (SLIC) algorithm [126], the transformed disparities were grouped into super pixels, and potholes were detected by identifying super pixels with intensities below an adaptive threshold. In experimental results, the proposed algorithm has been demonstrated to be highly accurate and efficient in road pothole detection, achieving 0.98 detection rates and an F1-score of 0.89. The stereo vision-based system presented a significant advance in road damage detection methodologies, which reconstructed the road surface in 3D and detected and characterized potholes efficiently.

By applying the Haar Wavelet Transform (HWT) to accelerometer signals, Silveira Rodriguez et al. (2022) [127] presented an automated system for detecting potholes. Due to the advent of embedded vehicle technology and the impending introduction of autonomous vehicles, road condition monitoring is becoming increasingly important. The proposed methodology capitalized on the advantages of low-cost processing in both signal acquisition and analysis stages. A two-step threshold procedure was used to analyze wavelet coefficients, allowing potholes to be detected as substantial variations in the accelerometer data. Adaptive threshold estimation eliminated manual calibration and identified standard signal patterns associated with acceptable road conditions. An actual vehicle was used to demonstrate the efficiency of the proposed methodology in addition to a controlled environment scenario using a robot car. Based on the number of steps assumed in the threshold procedure, the study highlighted the potential of the two-step threshold procedure for detecting potholes. The procedure can also be applied to automatic threshold operations and recognizing road conditions. As a result, the Haar Wavelet Transform method used in signal processing proved to be an extremely robust tool for detecting potholes in controlled and real-world scenarios.

An efficient method based on unsupervised vision was developed by Akagic et al. (2017) [118] to automate the pothole detection process. This study addressed the crucial need for road safety by making timely repairs and maintenance. As part of the proposed methodology, 2D images were automatically analyzed to detect potholes, bypassing the need for training and filtering. The search for potholes was restricted to the defined area by manipulating the RGB color space and segmenting images. Using low-cost and efficient processes that did not require expensive equipment or extensive filtering, the method was designed to work optimally under fair weather conditions during the daytime. In order to detect potholes on asphalt pavement, cropped images were compared, and the Otsu thresholding method was employed, eliminating linear and image boundary shapes to isolate pothole regions. This methodology accurately extracted the region of interest to detect potholes within asphalt pavements. The method was tested on 80 pothole images and achieved a high detection rate, accurately detecting all potholes with a 0.82 surface estimation accuracy.

In another study, a 2D vision-based approach was presented by Ouma and Hahn (2017) [128] to detect and quantify incipient potholes on asphalt road pavements in urban areas. This study employed image segmentation techniques to address pothole detection as a clustering problem within mixed pixels. Moreover, the challenges posed by expensive 3D imaging and reconstruction methods were addressed in this study. For superpixel classification of pavement defects and non-defects, multi-scale texture-based filtering using a wavelet transform was integrated with the Fuzzy C-Means clustering (FCM) algorithm [129]. Further refinement was achieved through morphological reconstruction, which improved the accuracy of pothole detection and segmentation. This method was implemented and validated in MATLAB software using 75 experimental image datasets. According to evaluation metrics such as the dice coefficient, Jaccard Index, and sensitivity, pothole detection accuracy was high at 0.87, 0.77%, and 0.97%, respectively [129]. As a result of this method, background noise was reduced, images were smoothed, and pothole shapes and sizes were accurately estimated. Table 2 summarizes the reviewed studies that used 2D image analysis for pothole detection.

### 4.2. Three-Dimensional Point Cloud Processing

In the case of 3D road point cloud processing, the most used methodologies often consist of a two-stage pipeline [131]. In the initial stage, the observed 3D road point cloud experiences a transformative process, wherein it is interpolated into a tangible geometric model. This model, frequently represented by planar or quadratic surfaces, is a structured abstraction of the complex spatial information inherent in the raw data. Subsequently, in the second stage, the processed 3D road point cloud is subjected to segmentation, a critical process involving a meticulous comparative analysis with the previously interpolated geometric model. This segmentation process is crucial in determining and isolating different features and patterns within the observed data, contributing to a more nuanced understanding of the road environment. For instance, Wu et al. (2021) [132] introduced a novel scale-adaptive framework for detecting and tracking road potholes. In their proposed methodology, a quadratic surface was initially fitted to the 3D road point cloud, generated using the Global Polynomial Transformation-Semi-Global Matching (GPT-SGM) algorithm. Notably, the surface modeling process incorporated crucial everyday vector information obtained by applying the three-filters-to-normal (3F2N), an ultra-fast and accurate surface average estimator. The comparison between the actual and modeled 3D road surface point clouds enabled the extraction of pothole point clouds. The robustness of this innovative road pothole detection and tracking framework was substantiated through extensive experimental results, both qualitatively and quantitatively.

In another study, Zhang and Elaksher (2012) [105] introduced a cutting-edge unmanned aerial vehicle (UAV)-based digital imaging system for the efficient collection of surface condition data along rural roads. Departing from conventional approaches, the method employed aerial assessment, using imagery collected from an unmanned platform to construct a 3D surface model specifically focused on road distress areas for accurate measurements. This system integrated a cost-effective model helicopter with a digital camera, a Global Positioning System (GPS) receiver, an Inertial Navigation System (INS), and a geomagnetic sensor. A new image processing algorithm was then developed to provide the precise orientation of acquired images, enabling the generation of detailed 3D road surface models and ortho images. This capability facilitated accurately measuring the size and dimensions of areas of road surface distress. Experimentation results highlighted the system’s performance, demonstrating high accuracy and reliability. Evaluation against known dimensions through 2D and 3D models showed sub-centimeter measurement accuracy.

To address the need for proactive approaches to pothole detection during driving scenarios, Li et al. (2018) [133] introduced a stereo vision system engineered to enable drivers with an advanced understanding of road conditions. The system was designed around two USB cameras, synchronously acquiring images to facilitate an accurate assessment of the road environment. Then, the parameters were collected through camera calibration with a checkerboard and used to compute the disparity map, enabling the projection of 2D image points into 3D world points. A bi-square-weighted, robust least squares approximation was applied to fit a road surface model using all 3D points to determine potholes. Consequently, points falling below this model were identified as the pothole region. The system further provides detailed information regarding the size and depth of each detected pothole.

Du et al. (2020) [134] developed an efficient method grounded in 3D point cloud segmentation. Using binocular stereo vision to acquire detailed 3D point clouds, the method initiated by fitting the pavement plane and removing it from the overall 3D point cloud representation of the road scene. This process allowed for the preliminary extraction of potholes within the scene. The method incorporated K-means [129] clustering and region-growing algorithms [27] to further refine and precisely delineate the potholes. The method’s ability to accurately detect potholes within complex scenes highlighted its potential as a practical and robust solution for automated pothole detection.

Using the mobile laser scanning point cloud data applications, extracting pavement damage information poses a significant challenge. Notably, the conventional approach of depending only on relative distance for pothole detection has produced incorrect results. Therefore, Ma et al. (2023) [135] introduced an advanced pothole detection method that integrates directed distance and skewed distribution to address this limitation. This method commences with the swift localization of potholes through directed distance calculations derived from the points and the locally fitted plane. Subsequent monomerization and denoising of potential potholes were achieved through density clustering. In this method, the potholes were determined using the negatively skewed distribution of the directed distance histogram, and the skewness coefficient plays a vital role in precisely determining potholes. Finally, rigorous experimentation conducted on the road with adverse conditions substantiates the efficacy and practicality of the proposed method. The results demonstrate its capacity for automatically detecting potholes with varying shapes and degrees of deformation. Table 3 summarizes the reviewed studies that used 3D point cloud processing for pothole detection.

### 4.3. Machine Learning and Deep Learning Approaches

As a subset of artificial intelligence (AI) technology, machine learning allows computers to learn and make decisions based on data without explicit programming. Machine learning algorithms are divided into supervised and unsupervised algorithms. In supervised learning, algorithms learn patterns and relationships from labeled data, whereas unsupervised learning extracts insights from unlabeled data [73]. Supervised algorithms, including advanced deep learning techniques like deep convolutional neural networks (DCNNs), have become integral in accurately detecting road potholes due to their ability to learn and identify complex patterns in data. Instead of explicitly setting parameters, DCNNs learn complex patterns and features through back-propagation, which is when the network adjusts its parameters based on annotated road data [138]. Image classification, object detection, and segmentation networks are the three most commonly used data-driven techniques for detecting road potholes. Image classification networks identify pothole-positive and pothole-negative road images, object detection networks identify potholes at the instance level, while semantic segmentation networks segment road images to detect potholes at the pixel or semantic level. Machine learning is a powerful tool that can detect road damage efficiently and effectively using these approaches [139].

**Figure 5:**
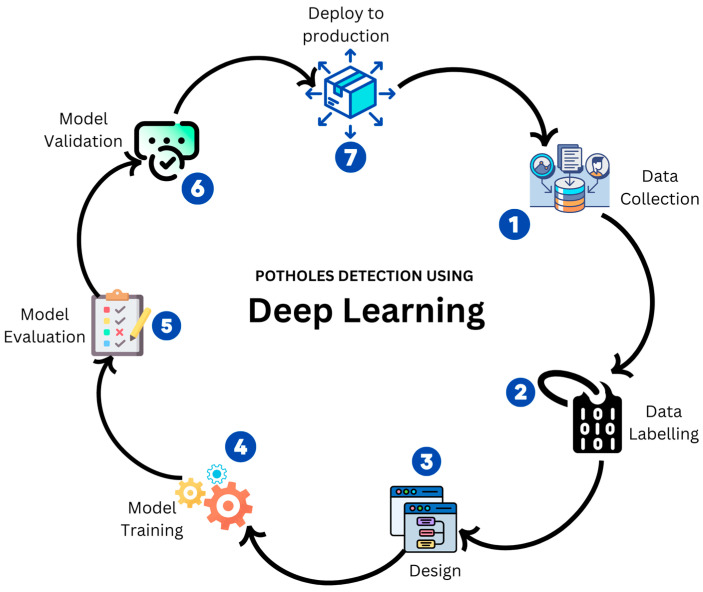
Workflow for pothole detection using deep learning.

Deep learning for pothole detection typically involves several key steps, including data collection, labeling, model designing, training, evaluation, and deployment (as illustrated in Figure 2). In order to facilitate the training and testing of the deep learning model, a substantial dataset comprising both images of potholes and images of non-pothole areas is collected. The images have been meticulously labeled, with bounding boxes drawn around the potholes and class labels assigned to indicate the interest areas. Next, an appropriate deep learning model, such as a CNN, is designed for the pothole detection task. The designed model is then trained on the training set and then learns patterns and features of the potholes. Subsequently, the trained model is evaluated on a separate test dataset to assess its efficacy in detecting potholes, as depicted in Figure 5. Upon successful evaluation, the trained model is deployed in real-world applications, such as road inspection systems, enabling real-time pothole detection.

Data collection for pothole detection is fundamental in training a deep learning model and involves several essential procedures. Initially, a diverse array of road images containing potholes and non-pothole regions is acquired using cameras or other imaging devices. These images are meticulously selected to filter out irrelevant or unsuitable samples. Subsequently, the selected images are labeled by annotators, with bounding boxes drawn around the potholes and corresponding class labels assigned. Data augmentation techniques, such as flipping, rotation, and scaling, are then applied to augment the labeled dataset, enhancing its size and diversity. Finally, the labeled data are split into training and testing datasets, with a portion reserved for evaluating the trained model’s performance.

Model designing plays a crucial role in determining the success of a pothole detection system, necessitating careful consideration of various factors such as accuracy, speed, computational complexity, and interpretability. Different deep learning models, each with distinct architectures and capabilities, are evaluated based on their performance on similar tasks. Popular models for object detection, such as Faster R-CNN [140], YOLO [141], and RetinaNet [142], are commonly considered for pothole detection tasks, with the final choice contingent upon the specific requirements and constraints of the application.

Model training involves fine-tuning the parameters of the designed deep learning model using the labeled data to optimize its performance for pothole detection. The labeled data are prepared by dividing them into training and validation sets and normalizing them to ensure consistency. The model architecture, loss function, and optimizer are defined, and the model is trained iteratively on the training data to minimize the loss function. Hyperparameters, such as learning rate and batch size, are fine-tuned to enhance the model’s performance. The trained model’s performance is evaluated on the validation set, and the best-performing model is selected for further refinement or deployment.

Model evaluation is crucial for assessing the trained model’s performance in accurately, reliably, and robustly detecting potholes. Various metrics, including precision, recall, F1-score, mean average precision (mAP), false positive rate (FPR), and false negative rate (FNR), are commonly employed to quantify the model’s performance. These metrics provide an understanding of the balance between correctly identified potholes and false detections. Additionally, visual inspection of the model’s predictions on the test data provides the accuracy and correctness of the detections, helping to identify any systematic errors. The evaluation results inform potential improvements to the model, such as collecting more training data or adjusting the model architecture and hyperparameters to enhance its overall effectiveness in real-world applications.

Finally, deployment entails making the trained deep learning model available for real-world use, involving several steps such as model optimization, integration with hardware, deployment environment setup, application programming interface (API) design, and ongoing monitoring and maintenance. A well-deployed model enables accurate and reliable pothole detection in real-time, facilitating timely interventions to repair roadways and enhance road safety.

#### 4.3.1. Object Detection

Pothole detection using object detection methods is classified into three types: Single Shot Multibox Detectors (SSDs), region-based CNNs (R-CNNs), and You Only Look Once (YOLO). These techniques have significantly advanced the ability to identify potholes within images. SSD integrates various image classification networks into a backbone network and an SSD head for effective pothole detection, as demonstrated in studies utilizing MobileNet [143], ResNet-34 [144], and RetinaNet [31] as backbone networks [109,145]. On the other hand, the R-CNN series, including Faster R-CNN variants, has shown remarkable performance in detecting road potholes, exceptionally Faster R-CNN with ResNet-101 as the backbone network, which outperformed its competitors [55,140]. Contrary to this, the YOLO series divides a road image into grids and generates bounding boxes to detect potholes. Studies using YOLOv2 [146], YOLOv3 [147], and YOLOv3 Tiny [147] have demonstrated successful road pothole detection, with YOLOv3 Tiny and YOLOv3 Spatial Pyramid Pooling (SPP) showing higher detection accuracy [148]. Each method has its unique approach, and YOLO is highly efficient, and SSD can detect multiple boxes. Although these object detection methodologies excel at instance-level predictions, pixel-level detection remains challenging [4].

The advancements in these methodologies represent substantial progress in identifying and managing road potholes. For instance, an innovative approach was introduced by Maeda et al. (2018) [109] to detect and classify road damage accurately. The team meticulously curated a substantial dataset of road damage images containing 15,435 instances of damage using a smartphone mounted on a vehicle captured in diverse weather and lighting conditions using image processing techniques and deep learning methodologies. Moreover, the dataset provided images and annotated bounding boxes that identified eight distinct types of road damage, verified by road administrators with their expertise. The model was trained and evaluated using this dataset and a CNN-based object detection method. Using a smartphone, the model achieved inference times of as little as 1.5 s for the most detectable damage categories, with high precision and recall rates exceeding 0.75. A smartphone app, trained models, source code, and the dataset were also made public, fostering accessibility and encouraging future research. Besides presenting a robust method for detecting road damage, this research also applied a foundation for widespread, cost-effective road inspection methods that can be applied in resource-limited regions.

As part of the road damage detection and classification challenge, Wang et al. (2018) [149] introduced a novel methodology for detecting road damage using images obtained from a smartphone mounted on a vehicle. They employed the Faster R-CNN framework, optimizing it by analyzing aspect ratios and sizes of damaged areas in their training dataset. Various data augmentation techniques, such as contrast transformation, brightness adjustment, and Gaussian blur, were implemented to mitigate imbalanced data distribution. The model achieved a mean F1-Score of 0.62 in the competition, outperforming the original Faster R-CNN model’s performance. The source code and model were made publicly available on GitHub, enhancing accessibility and advancing road damage detection methodologies.

In a study by Dharneeshkar et al. (2020) [148], a method was developed specifically for addressing the issue of potholes in countries like India, where manual road maintenance struggles to keep up with increasing accident rates caused by these hazards. They curated an annotated dataset of 1500 images of Indian roads to train CNNs using models like YOLOv3, YOLOv2, and YOLOv3-tiny. Despite the irregular shapes of potholes, their approach achieved effective detection with reasonable accuracy, as assessed by mAP, precision, and recall metrics. The study also proposed a practical implementation using Raspberry Pis [150] with cameras installed on vehicle dashboards to track pothole locations via GPS, enabling proactive maintenance strategies in regions facing similar challenges.

Yebes et al. (2020) [55] presented an automated approach to identify potholes across diverse global road scenes using advanced AI techniques. They used a variety of cameras and vehicles to collect images from cities worldwide and trained four DNNs for pothole detection. By achieving a mean average precision of over 0.75, their models were fine-tuned and evaluated in various environments, demonstrating robust performance. As part of the AUTOPILOT H2020 project, their system integrated successfully with the Nvidia DrivePX2 platform in actual vehicles, capable of providing road hazard warnings through Internet of Things (IoT) technology. Despite challenges like annotation errors and limited real-time performance, their approach highlighted significant potential for enhancing road safety and maintenance practices globally.

Gupta et al. (2020) [145] proposed an automated method for pothole detection using thermal imaging and deep neural networks to address the global issues caused by road potholes. They used modified ResNet34-SSD and ResNet50-RetinaNet models to localize potholes based on bounding boxes in thermal images. The ResNet50-RetinaNet model achieved a precision of 0.91 in pothole localization, marking a significant advancement. Thermal imaging enhances fault detection, particularly in challenging conditions like fog or night, ensuring reliable performance for timely road maintenance. The study highlighted the practicality and effectiveness of this approach in real-world applications, assisting authorities in prioritizing road damage repairs and mitigating accidents and injuries caused by potholes. Integrating accurate deep neural networks with cost-effective thermal imaging significantly enhances road safety and maintenance practices.

In their study, Saisree and U (2023) [15] tackled pothole detection on muddy and highway roads to prevent accidents. They developed a deep learning algorithm system to classify images from internet datasets depicting these road conditions. Pretrained models such as ResNet50 [151], InceptionResNetV2 [152], and VGG19 [153] were fine-tuned using specific datasets collected from muddy and Kaggle highway images. An evaluation through a web application demonstrated the system’s capability to distinguish roads with and without potholes based on accuracy, precision, and recall metrics. Notably, VGG19 outperformed ResNet50 and InceptionResNetV2 with accuracies of 0.97 for highway roads and 0.98 for muddy roads, showcasing its effectiveness across various road scenarios.

#### 4.3.2. Image Classification

Road pothole detection methodologies have evolved from traditional image processing techniques depending on support vector machines (SVMs) to more advanced DCNNs. Initially, SVM-based approaches utilized hand-crafted visual features, providing foundational understandings but needing assistance with scalability and performance as datasets expanded. In contrast, DCNNs have revolutionized pothole detection by learning hierarchical visual features autonomously. They excel in distinguishing between pothole and non-pothole road images with unprecedented efficiency. For example, Ye et al. (2019) [154] introduced a pioneering method using CNNs to detect and localize potholes in asphalt pavements. Their study used a comprehensive dataset of 96,000 pavement images to train and test two CNN models: a conventional CNN and a pre-pooling CNN variant. The pre-pooling CNN incorporated a preprocessing layer before the initial convolutional step, improving its ability to handle varying light conditions and pavement materials. During testing, the optimized pre-pooling CNN achieved a precision of 0.98. This approach signifies a paradigm shift from manual pavement inspection methods to autonomous ones. It offers superior precision, stability, and effectiveness over traditional image processing techniques by autonomously extracting pothole features and accurately determining their locations under diverse real-world conditions.

Gao et al. (2020) [155] introduced an innovative approach for detecting and segmenting potholes on cement concrete pavements using digital image analysis techniques. Their method employed an industrial camera setup and integrated several image processing steps, including texture filters, grayscale conversion, morphology, and connected domain extraction techniques. The study validated a machine learning model based on LIBSVM [155], a widely used algorithm for classification and regression, using data collected from agricultural and pastoral areas of Inner Mongolia, China. The model was specifically trained to differentiate between potholes, longitudinal cracks, transverse cracks, and complex cracks. The experimental results demonstrated good performance metrics for pothole recognition: 100% recall, 97.4% precision, and 98.7% F1-Score. Moreover, the study reported a high overlap rate of 76.8% between the extracted pothole regions and the original pavement images, with over 90% overlap. The proposed method exhibited superior segmentation effects and processing efficiency compared to alternative methods such as Otsu thresholding, edge detection, K-means clustering, and watershed techniques. However, the study acknowledged challenges in accurately detecting potholes covered by sandy soil, suggesting areas for future improvement in the detection system’s capabilities.

In another study, Aparna et al. (2022) [156] investigated the application of thermal imaging for improving pothole detection methodologies. Utilizing a thermal camera, they created a diverse dataset of pothole images to train CNNs, marking a novel advancement in this domain. The study explored self-built and pre-trained CNN models, assessing their efficacy in pothole detection through augmentation techniques. Notably, using a pre-trained CNN based on ResNet, the research set a new standard with 97.08% accuracy in pothole detection. This achievement underscores the potential of AI-driven thermal imaging systems, highlighting their benefits, such as heightened accuracy, cost-effectiveness, adaptability to adverse weather conditions, and reduced risk associated with physical detection methods. The study exemplifies how thermal imaging, empowered by CNNs, can revolutionize pothole detection methodologies.

In a separate investigation, Espindola et al. (2022) [157] focused on distress measurement within pavement management systems, mainly through the use of images obtained from right-of-way (ROW) video surveys. Their approach centered on employing CNNs for multi-label classification (MLC) of distress types, eliminating the need for precise distress location data within lanes. Using lightweight CNN architectures, including VGG16, ResNet-34, and ResNet-50, their MLC models achieved exceptional accuracy rates of up to 97% and an F1-score of 93% in identifying distress types such as potholes, cracks, patches, and bleeding. This method proved adaptable across various imaging hardware setups, showcasing its potential integration into network-level pavement management systems. The study highlights the efficiency and versatility of MLC techniques in pavement evaluation, offering cost and time savings compared to traditional methods and expensive sensor technologies like laser scanners.

Egaji et al. (2021) [158] developed an intelligent pothole detection system based on data gathered from mobile sensors. Using a 2 s non-overlapping moving window to pre-process the collected data, relevant statistical features that are crucial to the training of a binary classifier can be extracted. In a comparative study, five binary classification models were employed on balanced datasets to assess the efficiency of various machine learning models, namely naïve Bayes, Logistic Regression, SVM, KNN, and Random Forest Tree. Before feature extraction, the training and validation datasets were separated from the test dataset to eliminate similarity biases. A 2 s non-overlapping window was used during feature extraction to ensure data consistency across the training/validation and test sets. A stratified K-fold cross-validation technique with K = 10 was applied exclusively to the training dataset for model evaluation. In the test dataset, both Random Forest Tree and KNN models showed an accuracy of 0.88. Furthermore, the Random Forest Tree model’s performance was considerably enhanced after applying random search hyperparameter tuning, achieving significantly improved metrics with accuracy, precision, recall, and F-scores of 0.94, 1.0, 0.88, and 0.94, respectively. Despite achieving a perfect precision score, the recall has some limitations due to several false negatives in the model. The study emphasizes the need for a more extensive and varied dataset encompassing diverse road and vehicle types to improve the model’s overall performance and categorization capabilities.

Hoang et al. (2018) [159] introduced an innovative AI-based model to detect potholes on asphalt pavement surfaces. In order to extract pertinent features from digital pavement images, this study used image processing techniques such as Gaussian filters [159], steerable filters, and integral projections. A synergistic combination of these techniques contributed to the extraction of features. The Structure-from-Motion (SfM) created a robust pothole map as the Gaussian filtering (GF) was a denoising technique. An image processing approach was then employed to represent image features, particularly on pothole identification, numerically. A simple moving average technique was proposed to streamline the feature set, reducing the number of features from 300 to 60 to improve processing efficiency. Two AI approaches were employed using these image-derived features: artificial neural networks (ANNs) and least squares support vector machines (LS-SVMs). To train and validate the models, 200 image samples were used to categorize them as potholes and non-potholes. A repeated subsampling procedure over 20 runs confirms that both ANN and LS-SVM are effective at detecting potholes, with classification accuracy rates exceeding 85%. With an area under the curve (AUC) of 0.96, LS-SVM shows the highest classification accuracy rate of 89%. When combined with LS-SVM, the proposed AI-centric approach has immense potential for assisting road inspectors and transportation agencies in detecting pavement potholes more efficiently.

#### 4.3.3. Semantic Segmentation

Semantic segmentation is a vital image recognition task in computer vision and image processing. This technique assigns a specific class label to each pixel within an image, making it a fundamental method in image recognition [160]. Semantic segmentation has applications across various fields, including scene interpretation [161], medical image analysis [162], robotic perception [163], video surveillance [164], augmented reality [165], and image compression [166]. Due to its versatility and accuracy, deep learning-based semantic segmentation has become a powerful tool in the computer vision field [166]. In particular, semantic segmentation is essential for road scene segmentation, a critical component of autonomous vehicle systems [167,168,169]. Road segmentation using semantic segmentation significantly enhances road infrastructure in underdeveloped regions by effectively identifying road-related objects in images [170]. For example, Masihullah et al. (2021) [171] presented an attention-based coupled framework to segment roads and potholes in unstructured driving environments. By utilizing a continuous observation setup through a vehicle-mounted camera, this framework addresses the need for Advanced Driver Assistance Systems (ADASs) in areas with poorly defined or maintained drivable surfaces.

The key aspects of this work include presenting a unified approach for simultaneously segmenting roads and potholes specifically designed for unstructured environments. The framework enhances segmentation accuracy by integrating attention-based refinement with feature fusion. Additionally, the study explores few-shot learning for pothole detection, demonstrating improved accuracy with limited training samples, particularly using the Indian Driving Dataset (IDD). The framework demonstrated exceptional performance in road segmentation on both the Institute of Technology and Toyota Technological Institute (KITTI) and IDD datasets, achieving a remarkable mean intersection over union (mIoU) of 98.42% for road segmentation and 73.83% for pothole segmentation. The future extensions of this study encompass refining the model for diverse weather and lighting conditions and exploring semi-supervised learning techniques to enhance classification performance, particularly in cases where there is a significant disparity between labeled and unlabeled data distributions.

Previous studies addressed the critical task of segmenting roads and potholes as part of a road condition monitoring system. In order to create a community-based road monitoring system, Pereira et al. (2019) [172] proposed a semantic segmentation method using the well-known U-Net [172] deep learning technique. The model was trained on diverse images of roads capturing different road conditions. Experimental results demonstrate the effectiveness of the U-Net model, which achieves an accuracy of 97% and a mean intersection over union (mIoU) of 0.86. The proposed approach illustrates the adaptability of U-Net, which was initially designed for medical image segmentation, to successfully segment paved road and pothole images. In addition to contributing to the broader field of road infrastructure management, the findings suggest the potential application of this method by government authorities for comprehensive monitoring and evaluation of road conditions across diverse territories.

Fan et al. (2021) [70] developed a single-modal semantic segmentation-based approach to pothole detection in another study. A CNN extracted visual features from input images. In order to achieve better discrimination between potholes and unscathed road areas, the system used a channel attention module to reweigh channel features across multiple feature maps. The potholes were then distinguished from their surroundings using an atrous spatial pyramid pooling module, which combined atrous convolutions with progressive dilation rates. The proposed multi-scale feature fusion module (MSFFM) enhanced semantic prediction by merging low-level information at boundaries where pixel categorization was complex. The system minimized interference and improved semantic segmentation results by reweighing and harmonizing feature maps across layers and channels. Based on the Pothole-600 dataset, the methodology demonstrated state-of-the-art performance in RGB and transformed disparity images, surpassing existing single-modal semantic segmentation networks. By integrating global and detailed information in road imagery, this novel approach bridged the semantic gap between different feature map layers to detect potholes accurately.

In the case of the semantic segmentation-based approach, Mouzinho and Fukai (2021) [160] introduced a novel approach to address the challenges in detecting road surface damages and markings, focusing specifically on potholes, cracks, and markings on paved roads from images collected by a dashcam. The proposed method employed a hierarchical structure in semantic segmentation using two layers of layers in the segmentation process. As a first step, the first layer separated the paved roads from the rest of the image by classifying them as separate. A second layer then detected potholes, cracks, and markings within this identified road area. By focusing on the road region, this strategy avoided background features that could lead to mis-segmentation. Both layers of the model were trained independently by employing the U-Net architecture. The outputs of these hierarchical layers were combined during prediction to create a complete segmentation map by element-wise multiplication. Detecting road damages and markings improved significantly over ordinary non-hierarchical segmentation in F1-score and IoU. Requiring more computational time than non-hierarchical segmentation was a disadvantage of this hierarchical segmentation approach, making it less accurate in class detection. Nevertheless, the method’s strength lies in its ability to improve the accuracy of individual class detection and facilitate the development of specific methods that can be used to detect road damage more accurately.

In the domain of pothole detection, Fan et al. (2021) [173] categorized existing approaches into computer vision-based and machine learning-based methods, highlighting the difficulty of preparing large, well-annotated datasets for training CNNs. Computer vision methods have traditionally focused on 2D or 3D modeling and segmentation algorithms. However, machine learning approaches, particularly those based on CNNs, have achieved popularity, although they have faced challenges with dataset preparation due to the sporadic nature of potholes. The paper uses a new stereo vision-based dataset and disparity transformation algorithm to enhance the distinction between damaged and undamaged roads, addressing this gap. A benchmark for state-of-the-art CNNs trained on disparity and transformed disparity images is presented in this research, which evaluates a variety of CNNs for semantic segmentation. The study also introduced a new CNN layer that optimizes image feature representations for semantic segmentation, called the graph attention layer (GAL), motivated by graph neural networks (GNNs). Compared with existing CNNs, GAL-DeepLabv3+ [173] demonstrates superior pothole detection accuracy. The paper provides benchmarks, introduces the GAL, and presents an efficient CNN for pothole detection, with extensive experiments and public access to the code, dataset, and benchmarks, fostering further advancements in the field. Table 4 summarizes the reviewed studies that used machine learning models for pothole detection.

### 4.4. Hybrid Approaches

Detecting road potholes using hybrid methods involves combining classical 2D image processing techniques, 3D point cloud data, segmentation algorithms, and machine/deep learning techniques. These approaches have been studied in the last decade, showing state-of-the-art results. Using these hybrid strategies, road pothole detection is enhanced by combining the strengths of different algorithms. By selecting keyframes containing potholes and simultaneously reconstructing the road’s 3D geometry, some approaches reduce redundant computations by combining 2D image processing with 3D point cloud processing for efficient detection [175]. To detect pixels at the pixel level, other hybrid methods combine classical image processing with machine learning models, including naïve Bayes classifiers [176] and histograms of oriented gradients (HOGs) [177,178]. As a result of recent advances in deep learning models such as Mask R-CNN [179] and YOLOv2 [146], road pothole volume and depth distribution are now more accurately detected and analyzed [18]. By combining diverse approaches for a more robust detection framework, these hybrid techniques propose an acceptable performance for pothole detection. For instance, using a meticulously designed top-down framework, Yousaf et al. (2018) [180] presented an innovative computer vision-based method for accurately detecting and localizing potholes within asphalt pavements. The authors used a bag-of-words (BoW) approach to establish a visual vocabulary for pavement surfaces, utilizing the scale-invariant feature transform (SIFT). A Support Vector Machine (SVM) was employed to effectively train and test histograms of words extracted from pavement images. A graph-cut segmentation scheme was then proposed to locate potholes accurately in labeled images. With a diverse dataset containing a range of pavement scenarios, the study evaluates the proposed scheme’s subjective and objective efficacy. The experimentation results produced a remarkable 95.7% accuracy in determining pothole images, demonstrating remarkable precision and recall. A 91.4% accuracy level was achieved in the objective assessment of pothole localization, indicating its effectiveness.

A revolutionary stereo vision-based algorithm was introduced by Fan et al. (2020) [98] to detect potholes on road surfaces, addressing the limitations of existing methods. This algorithm achieved highly accurate and computationally efficient pothole detection through the combination of innovative disparity transformation and modeling techniques and efficient processing methods. It used a novel algorithm to transform dense disparity maps, enhancing the distinction between damaged and undamaged road areas. The thresholding method of Otsu combined golden section search (GSS) with dynamic programming (DP) to extract undamaged road regions more efficiently. Using least squares fitting (LSF) and surface normal integration, the study robustly modeled disparities in these extracted areas through random sample consensus (RANSAC). A pothole detection algorithm accurately detected potholes by comparing actual and modeled disparity maps. Three pothole detection datasets were presented, augmenting the scientific field of stereo vision. The experimental results highlighted the method’s unparalleled effectiveness and potential for real-world implementation in road maintenance and safety, with 98.7% successful detection accuracy and 99.6% pixel-level accuracy.

Li et al. (2016) [181] introduced an innovative method for detecting and measuring potholes, which significantly caused road pavement distress and led to expensive repair claims. The proposed approach combined 2D images with ground-penetrating radar (GPR) data to detect potholes more efficiently and precisely. A novel pothole detector was developed by analyzing distinctive patterns reflected by GPR signals reflected by potholes in images and GPR data to filter noise and enhance relevant clues indicative of potholes. Despite its challenges in distinguishing aggregated pavement defects that may worsen into severe potholes, this detector detected potholes from other pavement defects. When potholes were detected in GPR data, the method triggered image processing, excluding frames with no pavement defects, to save computational resources. The geometrical active contour model enhanced pothole shape extraction by integrating GPR and image data. With this approach, pavement management programs can be developed to extract pothole information such as location, shape, and depth. A precision of 0.94, a recall of 0.90, and an accuracy rate of 0.88 were demonstrated in the validation experiments.

Wu et al. (2019) [182] proposed a novel algorithm to automatically detect and extract road pothole distress based on mobile point clouds and images acquired from mobile mapping sensors. This advanced algorithm employed a three-step process: a state-of-the-art neural network was used for 2D candidate pothole extraction from images, a point cloud analysis was used for 3D candidate pothole extraction, and a depth analysis was used for final pothole identification. Using the distinct texture features of potholes and road surfaces, the algorithm built a robust training set for DeepLabv3+ using pothole and patch distress images, allowing for accurate pixel-wise segmentation and classification. As a result of this, the original road point cloud around the candidate pothole edge was classified into interior and exterior points based on the relationship between the mobile point cloud and images. The algorithm distinguished potholes from patches by fitting a road plane with exterior points and analyzing the depth distribution of interior points. In real and simulation cases, the method was validated for its accuracy in identifying potholes, extracting affected lanes, and assessing the safety of the road environment. Additionally, the simulation experiment confirmed the algorithm’s geometric precision in locating potholes, demonstrating its remarkable mean size accuracy. In contrast to traditional approaches, this method detected potholes at a much higher rate than traditional approaches, offering significant potential for road maintenance and emergency response. However, it is important to note that the algorithm’s performance may be affected by factors such as lighting conditions and road surface variations, which could be potential areas for improvement. A key innovation in the automated extraction of potholes was the integration of point clouds for precise edge and depth analysis, which provided a foundation for subsequent measurements and interventions in road maintenance processes. Table 5 summarizes the reviewed studies that used hybrid methods for pothole detection.

As seen in Table 6, while various techniques have been explored, each presents distinct challenges and opportunities. Traditional 2D image processing offers simplicity and cost-effectiveness but struggles with adverse lighting conditions and occlusions. Three-dimensional point cloud methods provide superior depth information but require substantial computational resources and complex sensor setups. Machine learning, particularly deep learning, has demonstrated high accuracy but necessitates extensive labeled data and training time. Hybrid approaches, combining multiple techniques, hold promise for improved robustness but introduce integration complexities.

The choice of sensing system significantly impacts pothole detection performance. Camera-based systems, though widely available and cost-effective, are vulnerable to adverse weather and lighting conditions. Lidar sensors offer high precision but come with higher costs and limited range. Radar systems can operate in challenging weather but may struggle with small pothole detection. The optimal sensing system depends on factors such as desired accuracy, cost constraints, and environmental conditions. Despite advancements, challenges persist in real-time processing, detection under varying road conditions, and seamless integration with road maintenance systems. Ensuring data privacy and security is crucial for public acceptance and deployment. To address these challenges, algorithm optimization is essential.

Feature engineering, focusing on relevant image or point cloud characteristics, can enhance detection accuracy while reducing computational load. Efficient model architectures, such as lightweight neural networks, can accelerate processing without compromising performance. Model optimization techniques like pruning and quantization can further streamline computations. Additionally, exploring approximate computing offers potential speedups but requires careful consideration of accuracy trade-offs.

By combining these strategies and addressing the inherent limitations of different sensing systems, researchers can develop robust and efficient pothole detection systems. Future research should focus on creating large-scale, diverse datasets, exploring innovative sensing technologies, and developing intelligent algorithms capable of adapting to various road conditions. Ultimately, the goal is to deploy reliable pothole detection systems that improve road safety and infrastructure management.

Table 7 presents a comparative analysis of average precision, recall, F1-score, and computational cost for the investigated pothole detection methods based on findings from the reviewed literature. As anticipated, hybrid methods generally exhibit superior performance metrics across the board. However, this enhanced accuracy comes at the expense of increased computational complexity and system integration challenges. Machine learning techniques, particularly deep learning, offer a compelling trade-off between performance and efficiency, making them suitable candidates for real-time applications. While 2D image processing methods demonstrate lower accuracy, their simplicity and low computational cost render them viable options for resource-constrained environments or preliminary detection stages in a near real-time system.

## 5. Future Research Directions

Despite significant advancements in vision-based pothole detection methods using computer vision and machine learning, several challenges and opportunities for further research remain. Addressing these issues could significantly enhance the effectiveness, accuracy, and efficiency of pothole detection systems, thereby improving road safety and infrastructure maintenance. Firstly, the integration of multi-sensor data is a promising area of research. By combining data from various sensors, such as LiDAR, radar, and infrared cameras, researchers can develop a more comprehensive understanding of road conditions. Each sensor type has its strengths and weaknesses; for instance, LiDAR can provide precise distance measurements, radar can detect objects in poor visibility conditions, and infrared cameras can identify heat patterns. By leveraging the strengths of these different sensors, future systems can achieve higher detection accuracy, even under challenging environmental conditions, such as low light or adverse weather.

Another critical area for future research is the development of algorithms capable of real-time processing on edge devices. Current pothole detection systems often rely on cloud-based processing, which can introduce latency and dependency on network connectivity. Edge computing allows data to be processed closer to the source, enabling immediate pothole detection and reporting. This capability is particularly important for applications in autonomous vehicles and smart city infrastructure, where timely maintenance responses are crucial. Developing lightweight yet powerful algorithms that can run on resource-constrained edge devices will be key to advancing real-time pothole detection.

The exploration of advanced deep learning architectures also presents a valuable research direction. While convolutional neural networks (CNNs) have been widely used in pothole detection, newer models like transformers could offer improved performance. These advanced architectures can handle larger datasets and more complex patterns, potentially leading to higher detection accuracies and better generalization across different types of road surfaces and pothole appearances. Additionally, hybrid deep learning models that combine the strengths of various architectures could be explored to further enhance detection capabilities. Moreover, reducing the reliance on large labeled datasets through semi-supervised and unsupervised learning (non-supervised) techniques could make pothole detection systems more adaptable and scalable. Semi-supervised learning, which uses a small amount of labeled data along with a large amount of unlabeled data, can significantly reduce the time and cost associated with data annotation. Unsupervised learning methods, which do not require labeled data, could also be employed to identify patterns and anomalies in road surfaces. Techniques like self-supervised learning, which leverages the data themselves to generate supervisory signals, could be particularly useful in improving model performance without extensive labeled datasets.

Adaptability to diverse road conditions remains a significant challenge for pothole detection systems. Roads vary widely in materials, textures, and conditions due to weather and usage patterns. Developing methods that can adapt to these variations is crucial for creating robust and reliable detection systems. Domain adaptation and transfer learning are promising techniques to address this challenge. By training models on a variety of road conditions and then fine-tuning them for specific environments, researchers can create systems that perform well across different contexts. In addition, conducting temporal studies to track road deterioration over time can lead to the development of predictive maintenance models. These models could forecast potential pothole formation, allowing for proactive maintenance strategies that prevent potholes from becoming serious hazards. By continuously monitoring road conditions and analyzing trends over time, researchers can identify early warning signs of deterioration and intervene before significant damage occurs.

## 6. Conclusions

This article reviews various methodologies and technologies employed in detecting potholes, a critical aspect of road infrastructure maintenance. Potholes pose significant risks to road safety, economic stability, and environmental sustainability. The combination of advanced computer vision and machine learning techniques in pothole detection offers a promising solution to these challenges.

Our comprehensive overview categorizes pothole detection algorithms into four main approaches: traditional 2D image processing, 3D point cloud processing, machine learning and deep learning approaches, and hybrid methods. Each category has its strengths and limitations, collectively representing a multifaceted strategy for improving the accuracy and efficiency of pothole detection. The use of deep learning architectures, particularly U-Net, has shown remarkable accuracy and efficiency in semantic segmentation tasks, adapted from medical imaging to road condition monitoring. These methods demonstrate high accuracy and reliability, achieving up to 97% accuracy and a mean intersection over union (mIoU) of 0.86, illustrating the potential of advanced deep learning techniques in addressing road maintenance challenges. The integration of channel attention modules and atrous spatial pyramid pooling further enhances the performance of these models, providing robust solutions for precise and timely pothole detection.

Despite these advancements, the preparation of large, well-annotated datasets remains a significant challenge that is critical for training effective convolutional neural networks (CNNs). Traditional computer vision methods have laid the groundwork, but the shift towards machine learning approaches requires comprehensive and diverse datasets to achieve optimal performance. The integration of global and detailed road information has been pivotal in bridging the semantic gap, resulting in improved detection accuracy. The findings of this review underscore the potential application of these advanced methods by governmental authorities for systematic and efficient monitoring of road conditions. Implementing these technologies can lead to enhanced roadway safety, reduced maintenance costs, and better infrastructure management, ultimately contributing to safer and more sustainable transportation systems. Accurate data collection and preprocessing are foundational to effective feature extraction and classification. In pothole detection, data collection involves using various sensors and imaging technologies, including laser scanners, Microsoft Kinect sensors, and multi-view geometry, which gather data such as surface topography, depth, and texture. Preprocessing techniques, such as noise removal, data alignment, and feature extraction, are then applied to the collected data to prepare it for further analysis.

Publicly available datasets are crucial for advancing research and development, as they provide the necessary resources for training and validating detection models. The datasets reviewed in this paper demonstrate the diversity and scale of data required to develop robust and reliable pothole detection systems. Rapidly detecting and repairing potholes is essential for maintaining road safety and infrastructure integrity. More precise and efficient pothole detection systems can be developed using advanced computer vision and machine learning techniques. This paper reviews the state-of-the-art technologies and underscores the importance of continued research and innovation in this field. Future research should focus on enhancing the accuracy and scalability of these detection methods, integrating real-time data processing, and addressing the challenges posed by diverse environmental conditions. Road maintenance practices can be significantly improved through such efforts, contributing to safer, more sustainable transportation networks.

## Figures and Tables

**Table 1:** Open-access road pothole detection datasets.

Dataset Name	Description	Train	Validation	Test	Access Link
Abhinav Kulshreshth’s Pothole Detection Dataset	Combines images sourced from Google and Kaggle, containing both Normal and Pothole images.	1167	108	136	Kaggle (https://www.kaggle.com/datasets/abhinavkulshreshth/pothole-detection-dataset; accessed on 15 August 2024)
Viren Baraiya’s Pothole Detection Dataset	Includes images of healthy roads and roads with potholes.	724	32	16	Kaggle (https://www.kaggle.com/virenbr11/pothole-and-plain-rode-images; accessed on 15 August 2024)
Pothole-600 Dataset	Provides color images and transformed disparity images using stereo-matching algorithms.	402	67	79	Google (https://sites.google.com/view/pothole-600/dataset; accessed on 15 August 2024)
Semantic Segmentation of Potholes and Cracks Dataset	Contains image–mask pairs for semantic segmentation.	3340	496	504	DeepLearning (http://deeplearning.ge.imati.cnr.it/genova-5G/ http://deeplearning.ge.imati.cnr.it/genova-5G/video/pothole-mix-videos/pothole-mix-rgb-d-overlay-videos-concat.html; accessed on 15 August 2024)
Large-Scale Pothole Detection Dataset	Created for instance-level pothole detection.	3777	759	628	Kaggle (https://www.kaggle.com/datasets/sovitrath/road-pothole-images-for-pothole-detection; accessed on 15 August 2024)
Liu et al. (2024) Dataset	It supports multiple-type distress detection in asphalt concrete pavement using various imaging types.	2057	514	642	Zenodo (https://zenodo.org/records/11638443; accessed on 15 August 2024)
Object Detection Pothole Dataset	Contains images for object detection with YOLO models.	3033	491	246	Universe (https://universe.roboflow.com/intel-unnati-training-program/pothole-detection-bqu6s/dataset/9; accessed on 15 August 2024)
Semantic Segmentation Datasets of Indian Roads	Annotated images for road, pothole, footpath, shallow path, and background classes.	2475	752	329	Kaggle (https://www.kaggle.com/datasets/eyantraiit/semantic-segmentation-datasets-of-indian-roads; accessed on 15 August 2024)
Japan Road Damage Detection Dataset	Contains road images capturing instances of road damage using a smartphone-mounted car setup.	7718	4630	3087	GitHub (https://github.com/sekilab/RoadDamageDetector; accessed on 15 August 2024)

**Table 2:** Reviewed 2D image processing-based studies.

Authors	Data	Method	Descriptions
Makone and Rathod (2002) [130]	Grayscale image	Mean shift-based filtering, an iterative process for edge detection	Applying a moving window to reduce speckle noise, enhances edge and texture preservation, and detects potholes based on size constraints from extracted road section images.
Koch and Brilakis (2011) [21]	Color image	Histogram-based thresholding, morphological operations, elliptic regression	Road pothole detection by segmenting damaged and undamaged road regions and comparing textures inside and outside ellipses.
Buza et al. (2013) [27]	Color image	Otsu’s thresholding, spectral clustering	Segmentation of road images and extraction of damaged road areas (potholes) using spectral clustering.
Ryu et al. (2015) [117]	Color image	Histogram-based thresholding, morphological filters, geometric properties	Processing road images with morphological filters, segmenting using thresholding, and extracting potential pothole contours based on geometric properties.
Schiopu et al. (2016) [115]	Color image	Histogram-based thresholding, geometric properties	Generation of road pothole candidates through thresholding and determination of potholes based on specific geometric properties.
Akagic et al. (2017) [118]	Color image	RGB color space manipulation, dynamic road pixel selection, comparison	Detection of road potholes by manipulating RGB color space, dynamic pixel selection, and comparison based on previous methods.
Wang et al. (2017) [123]	Grayscale image	Wavelet energy field, morphological filters, Markov random fields	Construction of wavelet energy fields for highlighting road potholes, segmentation using Markov random fields, and refinement with morphological filters.
Chung and Khan (2019) [92]	Grayscale image	Otsu’s thresholding, morphological filters, distance transform, watershed algorithm	Segmentation of road images with Otsu’s method, morphological filtering, distance transform, and watershed algorithm for pothole detection.
Fan et al. (2019) [116]	Transformed disparity image	Disparity image transformation, Otsu’s thresholding, SLIC	Transformation of dense disparity images, segmentation using Otsu’s method, and detection of road potholes by grouping superpixels with lower values than a threshold.
Fan et al. (2022) [32]	Disparity map, color image	Stereo rig roll angle adjustment, semi-global matching, disparity map transformation, SLIC, adaptive thresholding	Innovative algorithm for pothole detection using stereo vision. Enhances perspective transformation accuracy and minimizes complexity by including stereo rig roll angle. The method uses semi-global matching for dense sub-pixel disparity maps, transforms disparities, and detects potholes with SLIC and adaptive thresholding. Achieved 0.98 detection rate and F1-score of 0.89.
Silveira Rodriguez et al. (2022) [127]	Accelerometer signals	Haar Wavelet Transform (HWT), two-step threshold procedure, adaptive threshold estimation	Automated pothole detection using HWT on accelerometer signals. Includes a two-step threshold procedure for detecting significant variations and adaptive threshold estimation to eliminate manual calibration. Demonstrated with real vehicle and robot car scenarios.

**Table 3:** Reviewed 3D point cloud-based studies.

Authors	Data	Method	Descriptions
Zhang and Elaksher (2012) [105]	Aerial imagery, 3D surface model	UAV-based digital imaging, 3D surface modeling, image processing algorithms	A UAV-based system using digital imagery to construct 3D surface models and ortho images for accurate measurement of road distress. High accuracy and reliability with sub-centimeter measurement accuracy.
Zhang (2013) [136]	3D point cloud	Stereo vision, quadratic surface fitting, connected component labeling (CCL)	A quadratic surface is fitted to the 3D road point cloud, and 3D points under the fitted surface are identified as potential potholes. Uses connected component labeling to isolate and identify potholes.
Li et al. (2018) [133]	Stereo images, disparity map	Stereo vision system, disparity map computation, road surface modeling	Stereo vision system using synchronized USB cameras to compute disparity maps and fit a road surface model. Potholes are identified based on points falling below this model, providing detailed information on size and depth.
Du et al. (2020) [134]	3D point clouds	Binocular stereo vision, plane fitting, K-means clustering, region-growing algorithms	The method used is binocular stereo vision to acquire 3D point clouds, fit a pavement plane, and remove it to extract potholes. Refines pothole detection with K-means clustering and region-growing algorithms for accurate delineation.
Wu et al. (2021) [132]	3D road point cloud	Quadratic surface fitting, Global Polynomial Transformation-Semi-Global Matching (GPT-SGM), three-filters-to-normal (3F2N)	Scale-adaptive framework for detecting and tracking road potholes using quadratic surface fitting and comparison with actual 3D point clouds. Incorporates 3F2N for accurate surface estimation, validated through extensive experimental results.
Ma et al. (2023) [135]	Mobile laser scanning data	Directed distance calculations, density clustering, skewed distribution analysis	Advanced pothole detection integrating directed distance and skewed distribution. Uses density clustering for monomerization and denoising, as well as skewness coefficient for precise pothole determination. Effective in detecting potholes with varying shapes and deformation.
Chen et al. (2024) [137]	UAV imagery, 3D point cloud	UAV-based imagery, slicing-based method, Pavement Pothole Detection Algorithm	Low-cost UAV-based method for automatic road pavement inspection. Converts imagery into 3D point clouds and applies a slicing-based algorithm. Achieves 0.01 m accuracy in pothole depth detection and maximum errors of 0.0053 m^3^ in volume evaluation.

**Table 4:** Reviewed machine learning-based studies.

Authors	Data	Method	Descriptions
Syed et al. (2021) [79]	Color images	Faster R-CNN	Developed a generalizable pothole detection model using Faster R-CNN, tested across multiple datasets with varied conditions (e.g., lighting, image size). Achieved accuracies between 70% and 90%.
Shaghouri et al. (2021) [174]	Color images	SSD-TensorFlow, YOLOv3-Darknet53, YOLOv4-CSPDarknet53	Deployed and tested different deep learning architectures to detect potholes in road images. YOLOv4 achieved the best performance with 81% recall, 85% precision, 85.39% mAP, and a processing speed of 20 FPS.
Saisree and U (2023) [15]	Images from internet datasets	Pre-trained CNN models (ResNet50, InceptionResNetV2, VGG19), fine-tuning	Developed a deep learning algorithm system for pothole detection on muddy and highway roads. Fine-tuned pre-trained models using datasets from muddy and Kaggle highway images. VGG19 outperformed ResNet50 and InceptionResNetV2, achieving accuracies of 0.97 for highway roads and 0.98 for muddy roads, demonstrating high effectiveness across various road conditions.
Park et al. (2022) [14]	Color images	YOLOv4, YOLOv4-tiny, YOLOv5	Applied three YOLO models for automated pothole detection using a dataset of 665 pothole images. The models were trained and validated until the loss function reached a steady state, and their performance was evaluated using mean average precision at a 50% intersection-over-union threshold (mAP@0.5). YOLOv4-tiny showed the best performance with a mAP@0.5 of 0.787, outperforming YOLOv4 and YOLOv5s. The study highlighted limitations in detecting small potholes at a distance and under adverse weather and lighting conditions.
Wanli Ye et al. (2019) [154]	Color image	Pre-pooling CNN	Developed a method for automated pothole detection using a pre-pooling CNN with 96,000 small images cropped from 400 raw color pothole images collected under different light conditions. The pre-pooling layer, inserted before the first convolution layer, improved precision compared to conventional CNNs. The method showed robustness to varying light and pavement conditions and demonstrated higher suitability for pothole detection than traditional methods like Sobel edge detection and K-means clustering analysis.
Aparna et al. (2022) [156]	Thermal images	Pre-trained CNN (ResNet), self-built CNN, data augmentation	Utilized thermal imaging to create a diverse dataset of pothole images. Trained CNNs, including a pre-trained ResNet model, achieving 97.08% accuracy in pothole detection. Demonstrated the advantages of AI-driven thermal imaging systems in terms of accuracy, cost-effectiveness, adaptability to adverse weather, and reduced physical detection risks.
Espindola et al. (2022) [157]	ROW video survey images	CNNs (VGG16, ResNet-34, ResNet-50) for multi-label classification	Employed lightweight CNN architectures for multi-label classification of pavement distress types, achieving up to 97% accuracy and 93% F1-score. The method proves adaptable across various imaging hardware setups, offering significant cost and time savings compared to traditional methods and expensive sensor technologies.
Egaji et al. (2021) [73]	Mobile sensor data	Naïve Bayes, Logistic Regression, SVM, KNN, Random Forest Tree, 2-s non-overlapping moving window, stratified K-fold cross-validation	Developed an intelligent pothole detection system using mobile sensor data, employing five binary classification models. Random Forest Tree and KNN models showed an accuracy of 0.88, with Random Forest Tree achieving significantly improved metrics (accuracy 0.94, precision 1.0, recall 0.88, F-score 0.94) after hyperparameter tuning.

**Table 5:** Reviewed studies that used hybrid approaches.

Authors	Data	Method	Descriptions
Salaudeen and Çelebi (2022) [183]	RGB images (low-resolution and super-resolution)	ESRGAN, YOLOv5, EfficientDet	Applied ESRGAN for generating super-resolution images and YOLOv5/EfficientDet for detecting potholes. Demonstrated improved performance in detecting small and distant potholes under various challenging conditions compared to state-of-the-art methods. YOLOv5 showed faster training and inference speeds.
Li et al. (2016) [181]	2D images and GPR data	Ground-penetrating radar (GPR), image processing, geometrical active contour model	Combined 2D images with GPR data to detect potholes. Developed a novel pothole detector by analyzing GPR signals reflected by potholes. Triggered image processing when potholes were detected in GPR data to save computational resources. The geometrical active contour model enhanced pothole shape extraction by integrating GPR and image data. Achieved a precision of 0.94, a recall of 0.90, and an accuracy rate of 0.88.
Fan et al. (2020) [98]	Stereo images, Disparity maps	Disparity transformation, Otsu thresholding, GSS, DP, LSF, RANSAC, surface normal integration	Used Otsu thresholding with GSS and DP to extract undamaged road regions. Employed LSF and surface normal integration with RANSAC for robust disparity modeling. Compared actual and modeled disparity maps for accurate pothole detection. Achieved 98.7% detection accuracy and 99.6% pixel-level accuracy.
Acharjee et al. (2020) [184]	Grayscale images	CNN, Gaussian filter, bilateral filter, median blur, Canny edge detection, dilation, erosion, contour detection	The input road segment image is converted to a grayscale version, then applied with a Gaussian filter to reduce noise. A bilateral filter and median blur are used to preserve edges. Canny edge detection finds edges, and dilation merges undesirable edges. Erosion and contour detection categorize potholes based on size constraints.

**Table 6:** General categories for pothole detection approaches.

Method	Key Features	Strengths	Weaknesses
2D Image Processing	Edge detection, texture analysis	Simplicity, low cost	Sensitivity to lighting conditions
3D Point Cloud Analysis	Depth measurement, surface reconstruction	High accuracy, detailed surface information	High computational cost, complex setup
Machine Learning	Feature extraction, classification	Adaptive, high detection rates	Requires large labeled datasets, training time
Hybrid Methods	Combination of the above methods	Enhanced accuracy, robustness	Increased complexity, integration challenges

**Table 7:** Average precision, recall, F1-score, and computational cost for the investigated pothole detection methods based on findings from the reviewed studies.

Method	Precision	Recall	F1-Score	Computational Cost
2D Image Processing	75%	70%	72%	Low
3D Point Cloud Analysis	90%	85%	87%	High
Machine Learning	85%	80%	82%	Medium
Hybrid Methods	92%	88%	90%	High

## Data Availability

Data are contained within the article.
